# Sources, Distribution, and Health Risks of Heavy Metal Contamination in the Tongren Mercury Mining Area: A Case Study on Mercury and Cadmium

**DOI:** 10.3390/toxics13070527

**Published:** 2025-06-23

**Authors:** Shuo Wang, Yani Guo, Huimin Hu, Yingqi Liang, Kun Li, Kuifu Zhang, Guiqiong Hou, Chunhai Li, Jiaxun Zhang, Zhenxing Wang

**Affiliations:** 1 School of Environmental and Chemical Engineering, Xi’an Polytechnic University, Xi’an 710600, China; wangshuo_202209w@163.com (S.W.); guoyani2002@163.com (Y.G.); 2 State Environmental Protection Key Laboratory of Water Environmental Simulation and Pollution Control, South China Institute of Environmental Sciences, Ministry of Ecology and Environment, Guangzhou 510655, China; huimin_hu2022@163.com (H.H.); lyq0708316@163.com (Y.L.); 3 Guangzhou Huacai Environmental Protection Technology Co., Ltd., Guangzhou 511480, China; nathan21@163.com (K.L.); 17285648554@163.com (K.Z.); 19970951970@163.com (J.Z.); 4 Chenzhou Tungsten Products Branch of Hunan Shizhuyuan Nonferrous Metals Co., Ltd., Chenzhou 423037, China; houguiqiong2025@163.com (G.H.); lichunhai2025@126.com (C.L.)

**Keywords:** soil, heavy metals, source apportionment, healthy risk assessment

## Abstract

This study assessed heavy metal contamination and associated health risks in soils and crops in the vicinity of a mercury mine located in Tongren, Guizhou Province, China, focusing on mercury (Hg), cadmium (Cd), arsenic (As), lead (Pb), and chromium (Cr). The study used the Index of Geological Accumulation (Igeo) and Health Risk Assessment (HRA) to quantify the level of contamination and assess the potential risks. The results showed that Area I was the most severely contaminated, with 94.24% of the sample sites being heavily contaminated with mercury, followed by Area II and Area III with severe cadmium contamination. The health risk assessment showed that children were exposed to non-carcinogenic risks of mercury and cadmium that exceeded the safety thresholds, with mercury being the major non-carcinogenic factor, especially through oral intake. The study also assessed the contribution of each heavy metal to pollution, with mercury contributing the most to ecological and health risks, especially in Areas I and III. The study highlights the urgent need to strengthen pollution control strategies, focusing on mining activities and agricultural inputs, to reduce risks and protect public health.

## 1. Introduction

While driving economic growth, the extraction and development of mineral deposits pose significant threats to the surrounding environment [1,2,3]. Primary pollutants include slag overflow from mining areas, emissions from smelting, and heavy metal contamination in acid mine water, which contributes to the pollution of both terrestrial and aquatic environments through rainfall runoff and deposition from the atmosphere [4]. The practice of randomized slag disposal causes heavy metals to accumulate in the soil, while smelting dust emissions worsen air quality and accelerate environmental degradation [5]. Identifying and evaluating the potential health risks associated with heavy metals in polluted soils is crucial for effective pollution management. The elements Hg, Cd, As, Pb, and Cr are closely studied because of their high toxicity, ability to bioaccumulate, and persistence in the environment [6]. The 2014 National Soil Pollution Survey Bulletin [7] indicates that heavy metal pollution affects areas of soil within China’s industrial and agricultural sectors, causing a decline in the extent of usable farmland. The exceedance rate of soil near contaminated enterprises is 36.3%, while that of arable land is 19.4% [8]. The accumulation of toxic metals within terrestrial soil systems adversely impacts both biological organisms and human health [9]. Chronic ingestion of heavy metal-laden foods may constitute a major health threat, heightening the likelihood of cancer, bone fractures, and neurological damage. It can also potentially cause abnormalities in cholesterol levels, reduced fertility, and immune system dysfunctions [10].

To mitigate soil contamination and its effects on human health, it is essential to thoroughly evaluate heavy metal patterns, source identification, and health risk assessment in industrially polluted areas. The combination of individual pollution indices (Pi), Nemero Integrated Pollution Index (NIPI) [11], and Potential Ecological Risk Index (RI) [12] is more suitable for evaluating soil contamination by heavy metals. The identification of heavy metal sources can be achieved through various source analysis methods [13], with correlation analysis (CA) serving as a preliminary tool to reveal the relationships between different heavy metals and their potential common sources. Additionally, the PMF is a receptor model and mathematical approach recommended by the USEPA for identifying the sources of pollutants, including heavy metals. For example, studies have shown that agricultural activities are the main anthropogenic source and that heavy metal accumulation is influenced by atmospheric deposition [14], natural sources, industrial activities, and transport sources [15,16]. Therefore, applying PMF modeling is essential for identifying the sources of heavy metals in soil. However, the assessment results of soil and crop contamination are not always positively correlated with health risks due to factors such as soil physicochemical properties and environmental influences. For example, in the literature, soil heavy metal concentrations did not exceed screening values, but consuming these crops posed high health risks [17,18]; conversely, crops grown in highly contaminated soils did not show significant health threats [19,20].

Building on this foundation, the present study explored soil and crop contamination near a mercury mining region in Southwest China, spanning three sectors: Area I, Area II, and Area III. Heavy metals such as Hg, Cd, As, Pb, and Cr were assessed in soils and crops. Soil and paddy rice contamination was assessed using individual pollution indicators and a combined pollution index to evaluate the potential risks of heavy metal contamination in agricultural soils. Additionally, source apportionment was optimized by coupling CA and PMF models. A health risk evaluation was conducted for both children and adults, considering multiple exposure pathways and the dietary intake of paddy rice.

This study aims to assess the environmental pollution status and potential health risks in the areas surrounding the mercury mine, filling the research gap in pollution exposure and health risk assessment in this region. Given that no systematic health risk assessment has been conducted in this area, this study analyzes the Geo-accumulation Index (Igeo) and the Potential Ecological Risk Index (PERI) to reveal the characteristics of heavy metal contamination in the soil and further evaluate its impact on the ecosystem and human health.

## 2. Materials and Methods

### 2.1. Study Area

**Figure 1:**
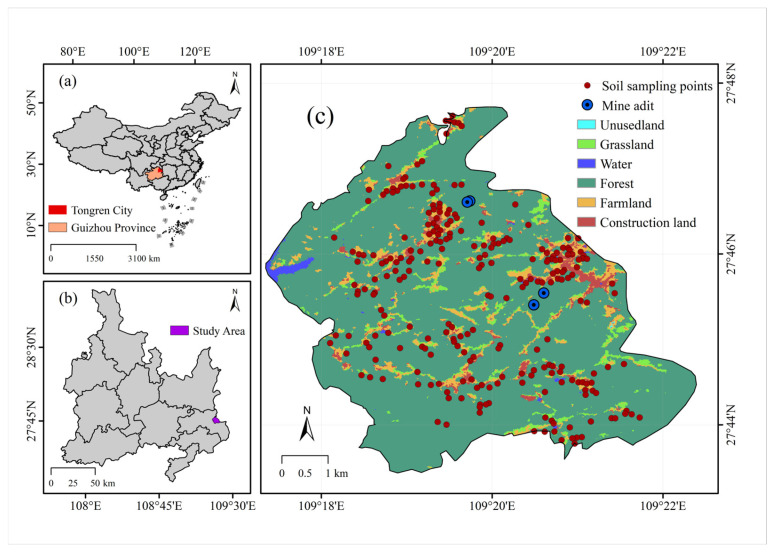
Map of the study area. (**a**) China Map, (**b**) study area, (**c**) Land use types and location of mines in the study area.

Bijiang, the central urban area of Tongren in northeastern Guizhou, lies at the junction of the Guizhou Highlands and the Western Hunan Hills, flanked by Fanjing Mountain and Xuefeng Mountain, with a total area of 1012 square kilometers. Guizhou lies within the Pacific Rim Mercury Mineralization Belt, boasting a mercury reserve of 88,000 tons, which constitutes 80% of the national total. It serves as a significant industrial hub for mercury production in China. Guizhou’s 630-year engagement in mercury extraction and smelting has been accompanied by its richness in lead, zinc, and numerous other minerals. In addition, the region has metal smelters, chemical plants, mercury waste treatment facilities, agricultural production, and transport infrastructure. Preliminary investigations indicate that the parent material for soil formation is sandstone weathering products, residual slope deposits, and sediments. The soil types are mainly red, yellow, and rice soils. Figure 1 illustrates the location and spatial distribution of the sampling locations.

### 2.2. Sampling and Analysis

Sample collection was performed following the principles of randomness. In total, 249 surface (0–20 cm) soil samples (52 from Area I, 97 from Area II, and 100 from Area III) were sampled. Soil specimens were obtained through the five-point sampling approach, securely sealed in polythene bags to avoid contamination, and transported to the laboratory for comprehensive testing. Soil specimens were subjected to natural air drying, fine grinding, sieving with a 100-mesh nylon screen, and homogenization and were then placed into transparent polyethene bags for storage. Soil Pb and Cd were extracted by HF-HCl-HNO_3_-HClO_4_ tetra-acid digestion method using a microwave digestion apparatus, heated to 180 °C, and maintained for 45 min. Soil Hg and As were extracted by aqua regia extraction, using hot plate digestion, placing the sample in a fume hood, heating to 95–105 °C, avoiding boiling, and maintaining the heating for 2–4 h until the solution became clear and transparent. Soil Cr was extracted by HF-HCl-HNO_3_ -HClO_4_ extraction using a microwave ablator and heated at 150–180 °C until the sample was completely ablated, and the solution was clear and transparent. Soil Cd, Pb, and Cr concentrations were determined using inductively coupled plasma mass spectrometry (ICP-MS; Thermo Scientific X7, Waltham, MA, USA), while Hg and As levels were assessed by atomic fluorescence spectrometry (AFS; Beijing Jitian Instruments AFS-820, Beijing, China). Cr (VI) was determined using alkaline extraction followed by flame atomic absorption spectrophotometry (TAS-990AFG, Beijing Purkinje General Instrument Co., Ltd., Beijing, China), with a detection limit of 0.5 mg/kg.

To ensure the accuracy and reliability of the soil heavy metal analysis results, this study strictly implemented quality assurance and quality control measures throughout the sample collection, processing, and experimental analysis stages. In the experimental analysis, nationally certified standard analytical methods were used for the determination of heavy metals. To assess the quality of the analysis, blank samples were included in each batch to check for any exogenous contamination during the analysis process. Additionally, parallel samples were run to evaluate analytical reproducibility, with deviations of results controlled within ±10%. National soil standards were used as standard reference materials to ensure the accuracy of the determination results. To further confirm the precision of the measurements, recovery checks were performed using certified reference materials (CRM), and the recovery rates were consistently within the acceptable range, which is between 90% and 110% for all analyzed metals. For quantification, all metal elements were analyzed using multi-point standard curves, and the correlation coefficients (R^2^) of these curves were all greater than 0.999, ensuring the reliability of the quantitative results.

### 2.3. Evaluation of Heavy Metal Pollution

(1) The Geo-accumulation Index (Igeo) was proposed by Müller in 1969 as a method for assessing the level of heavy metal contamination in soils. This index reflects the degree of heavy metal pollution by comparing the heavy metal content in soil samples with the background values (i.e., the natural levels in unpolluted areas). The higher the Igeo value, the more severe the pollution, and conversely, a lower value indicates lighter pollution or no pollution.

(1) $$I_{geo}=\log_{2}\left[C_{s}^{i}/\left(K\times C_{n}^{i}\right)\right]$$

where I_geo_ is the Geo-accumulation Index; $C_{s}^{i}$ is the measured concentration of the i-th heavy metal, mg·kg^−1^. $C_{n}^{i}$ is the background value of the i-th heavy metal. K is a conversion factor used to eliminate variations in background values caused by differences in local rocks, with a value of 1.5. Categorization of the Geo-accumulation Index (Igeo) is shown in Table S1. The background values of Hg, Cr, As, Pb, and Cr in the region are 0.13, 0.4, 13.48, 33.57, and 98.98 in mg/kg [21,22].

(2) The Nemerow Composite Pollution Index Method (PN)

(2) $$PN=\sqrt{\frac{P_{max}^{2}+P_{ave}^{2}}{2}}$$

P_ave_ refers to the mean pollution index of all individual contaminants in the soil, Pmax denotes the highest pollution index among these contaminants, and PN signifies the overall combined index of heavy metal contamination in the soil.

The N.L. Nemerow Pollution Index approach was applied to evaluate the aggregate pollution level induced by multiple contaminants. To assess the pollution levels of two heavy metals in soils and crops, both the individual pollution indices and the Nemerow composite pollution index were calculated using Equations (2) and (3). According to the Standardization Administration of China’s “Environmental Quality Soil Contamination Risk Management (Trial) Regulations” (GB15618-2018) [23], screening and risk control values were utilized as soil quality assessment criteria for heavy metals. The assessment results were classified based on the “Technical Specifications for Environmental Soil Monitoring” (HJ/T 166-2004) [24] of the Standardization Administration of China, using relevant indicators (Table S2). The study applied a weighting method to quantify how heavy metals influence the composite pollution index, as derived from the formula provided below:

(3) $$PW_{i}=P_{i}\times {\left(\sum_{i=1}^{n=2}P_{i}\right)}^{-1}\times 100\%$$

PWi is the dimensionless contribution of Hg and Cd to the combined soil or crop contamination level.

### 2.4. Health Risk Assessment Methodology

To evaluate the human health risks associated with heavy metal contamination, both non-carcinogenic and carcinogenic impacts were analyzed using the Health Risk Index (HI) and Carcinogenic Risk Index (CR), as established by the U.S. Environmental Protection Agency (USEPA, 2011) [25]. Consumption, dermal absorption, and inhalation are the primary pathways of heavy metal exposure from soil, with oral ingestion being the predominant route for exposure through crops [26]. Health risks were evaluated through multiple exposure pathways to determine the contributions of non-carcinogenic heavy metals in soil and crops to both children and adults. The detailed information is as follows:

(1) Exposure calculations:

The daily exposure of heavy metals through multiple exposure routes was computed as follows:

(4) $${ADD}_{ing-soil}=\frac{C\times IngR\times EF\times ED}{BW\times AT}\times 10^{-6}$$

(5) $${ADD}_{derm}=\frac{C\times SA\times SL\times ABS\times EF\times ED}{BW\times AT}\times 10^{-6}$$

(6) $${ADD}_{inh}=\frac{C\times {IR}_{inh}\times EF\times ED}{PET\times BW\times AT}$$

${ADD}_{ing-soil}$, ${ADD}_{derm},\mathrm{and}\;{ADD}_{inh}$ denote the average daily exposure of adults through three pathways: ingestion, inhalation, and dermal absorption, respectively, mg·kg^−1^·d^−1^; C signifies the levels of heavy metal concentrations in the soil, mg·kg^−1^, and the measurements for additional variables are provided in Table S3.

(2) Non-carcinogenic Risk Index calculations:

(7) $$HI=\sum {HQ}_{i}=\frac{{LADD}_{i}}{R_{fd}}$$

The parameter values can be found in Table S4.

When the HI (Hazard Index) or HQ_i_ (Hazard Quotient) metric is below 1, the non-carcinogenic risks from exposure to Hg and Cd through soil and crops are considered minimal. Conversely, when both values exceed 1, this indicates that the acceptable threshold for non-carcinogenic health risk has been surpassed. Regarding the carcinogenic risk of heavy metals, neither the USEPA model nor the Technical Guidelines for Risk Assessment of Contaminated Sites supply a carcinogenicity factor for Hg. As a result, this study excluded Hg from evaluating carcinogenic risks.

(3) Methodology for calculating the Carcinogenicity Risk Index:

(8) $${CR}_{i}=ADD\times SF$$

(9) $${TCR}_{t}=\sum {CR}_{i}$$

CR indicates the carcinogenic risk, while SF refers to the slope factor for carcinogenicity, (kg·d)·mg^−1^. The CR benchmark of the guideline is 10^−6^, but according to the research of relevant experts, the carcinogenic risk of 10^−6^~10^−4^ is also acceptable.

(4) The contributions of single pollutants to non-carcinogenic and carcinogenic risks through oral-soil ingestion, inhalation, and dermal absorption were computed using the following equations:

(10) $${PHQ}_{i}=\frac{{HQ}_{i}}{HI}\times 100\%$$

(11) $${PCR}_{i}=\frac{{CR}_{i}}{TCR}\times 100\%$$

PHQ_i_ is the non-carcinogenic risk contribution of Hg and Cd and is dimensionless, and PCR_i_ is the carcinogenic risk contribution of both pollutants and is also dimensionless.

### 2.5. Positive Definite Matrix Factor Model (PMF)

The principle of the PMF model [23] is to decompose the original matrix Eik into two-factor matrices (A_ij_ and B_ik_) and a corresponding residual matrix (g_ik_) using a least-squares iterative approach [27]. Through iterative factorization, the PMF model determines the optimal A and B matrices that minimize the target function Q, which is defined in the equation below:

(12) $$X_{ij}=\sum_{k=1}^{p}\left(g_{ik}f_{kj}+e_{ij}\right)$$

where X_ij_ denotes the measurement matrix, g_ik_ represents the contribution matrix, and f_kj_ is the source component spectrum. The term eij corresponds to the residual value for each sample. Here, i, j, and k indicate the total number of samples, the number of chemical elements, and the number of source factors, respectively. The following equation provides the Q-value.

(13) $$Q=\sum_{i=1}^{n}\sum_{j=1}^{m}{\left(\frac{e_{ij}}{u_{ij}}\right)}^{2}$$

where it signifies the uncertainty of heavy metal j in sample I, based on the MDL. If the concentration C exceeds the MDL, the uncertainty is then calculated using both the concentration and the MDL, as follows:

(14) $$u_{ij}=\sqrt{{\left(\mathrm{Error}\;\mathrm{Fraction}\times \mathrm{concentration}\right)}^{2}+{\left(0.5\times MOL\right)}^{2}}$$

Metals with concentrations at or below the MDL have their uncertainty evaluated as follows:

(15) $$u_{ij}=5/6\times MDL$$

### 2.6. Statistics and Analysis of Data

Microsoft Excel 2016 and SPSS 2021 were used for data analysis, and OringinPro2021 was used for mapping. Heavy metal concentrations were spatially mapped through ordinary kriging interpolation in ArcGIS 10.2 (ESRI, Redlands, CA, USA). Source apportionment was performed with EPA PMF 5.0 to resolve the contributing emission factors.

## 3. Results and Discussion

### 3.1. Evaluation of Heavy Metal Pollution in Agricultural Soils

#### 3.1.1. Soil Pollution Index

Descriptive statistics of soil heavy metals in the study area are presented in Table 1. The proportion of contaminated sample points of soil heavy metals to all sample points in the study area is shown in Table 2.

Table 1 shows the descriptive statistical analysis data of soil heavy metal concentrations in the study areas. Using the screening values in the soil environmental quality standards as reference, soil Cr in Areas I, II, and III did not exceed the screening values, while Hg, Cd, As, and Pb exceeded the screening values to varying degrees, with the screening rates from high to low being Hg > Cd > As > Pb. The screening rates of elemental Hg in the three areas were, respectively, 96.23%, 100%, and 97%, and the highest exceeding times were 363, 325, and 141 times, respectively. The mean values of the five heavy metals were 63.5 mg/kg, 55.52 mg/kg, 28.05 mg/kg, 13.09 mg/kg, and 0.73 mg/kg in Area I; 54.0 mg/kg, 55.2 mg/kg, 17.76 mg/kg, 23.68 mg/kg, and 0.52 mg/kg in Area II; and 61.9 mg/kg and 0.52 mg/kg in Area III, respectively. They were 61.9 mg/kg, 54.79 mg/kg, 21.43 mg/kg, 13.3 mg/kg, and 1.09 mg/kg, respectively, with Hg, Cd, and Pb being higher than the regional soil background values, indicating that the heavy metals all accumulated in the soil to some extent. The mean values of soil Pb in Area II, Area I, and Area III were 1.64, 1.65, and 1.63 times of the background values; the mean values of Cd were 1.3, 1.825, and 2.725 times of the soil background values; and the mean content of Hg was 182, 215, and 164 times the background values, which indicates that the accumulation effect of Hg was the most significant in the soil.

In this study, although total chromium concentrations in farmland soils ranged from 16 to 115 mg/kg, hexavalent chromium Cr(VI) was not detected. This outcome is likely attributed to the absence of chromium-related industrial pollution sources, as historical activities in the region were primarily focused on mercury mining, with no evident external input of chromium compounds. In soils, chromium predominantly exists in the trivalent form Cr(III), which is chemically stable and less toxic and is unlikely to oxidize into Cr(VI) under typical environmental conditions. The soils in the study area are generally neutral to slightly acidic and contain abundant organic matter and reducing agents, which suppress the formation and persistence of Cr(VI). Even if trace amounts of Cr(VI) were present, they would likely be reduced or adsorbed, falling below detection limits. The observed total chromium concentrations are within the range of natural background values, indicating minimal anthropogenic input. Taken together, the geochemical characteristics and environmental conditions reasonably explain the non-detection of hexavalent chromium.

**Table 1:** Descriptive analysis of soil heavy metal parameters in the study area.

Area	Element	Min/ (mg/kg)	Max/ (mg/kg)	Median/ (mg/kg)	SD/ (mg/kg)	CV/%	Exceedance Rate/ %
I	Hg	0.3	218.0	28.1	40.5	1.5	96.2
	Cd	0.1	3.4	0.7	0.6	0.8	77.4
	As	3.5	25.9	13.1	5.2	0.4	5.7
	Pb	13.0	85.0	55.5	14.6	0.3	3.8
	Cr(III)	32.0	82.0	63.5	11.9	0.2	0.0
II	Hg	0.7	195.0	23.7	37.5	1.6	100.0
	Cd	0.0	1.7	0.5	0.4	0.7	55.2
	As	4.9	46.5	17.8	8.1	0.5	19.8
	Pb	13.0	85.0	55.2	14.6	0.3	2.1
	Cr(III)	16.0	82.0	54.0	15.7	0.3	0.0
III	Hg	0.2	286.0	21.4	41.8	1.9	97.0
	Cd	0.1	6.6	1.1	1.1	1.0	86.0
	As	3.4	27.2	13.3	5.6	0.4	5.0
	Pb	28.0	140.0	54.8	16.4	0.3	0.0
	Cr(III)	47.0	115.0	61.9	10.7	0.2	0.0

The table data indicate significant variations in soil heavy metal contamination levels across the study areas, with Hg being the most severe pollutant. The proportion of heavily contaminated sample points reached 94.2% in Area I, 77.1% in Area II, and 92.0% in Area III, highlighting Hg as the primary pollution factor. Cd contamination was more prominent in Areas I and III, with the proportions of low, moderate, and heavy pollution sample points in Area I at 36.5%, 23.1%, and 17.3%, respectively, while in Area III, 42.0% of the sample points showed low pollution levels, indicating regional differences in Cd contamination. As and Pb exhibited relatively low contamination levels, remaining mostly at clean or warning levels, with Pb in Areas I and II predominantly classified as clean, whereas As in Area II had 18.6% of sample points at low pollution and 1.0% at moderate pollution levels, suggesting minor contamination risks. Cr remained almost entirely at clean levels in both Areas II and III, posing minimal contamination risk. Based on the comprehensive pollution index (PN), the proportions of heavily polluted sample points in the three regions were 90.4% in Area I, 72.2% in Area II, and 82.0% in Area III, further indicating that soil pollution in Areas I and III was more severe than in Area II. Overall, Hg emerged as the dominant pollutant across all areas, while Cd also showed significant contamination in certain regions, whereas As, Pb, and Cr presented relatively low pollution levels.

**Table 2:** Percentage of soil samples contaminated with heavy metals.

**Area**	**Elements**	**Percentage of Pi Samples/%**					**Percentage of PN Samples/%**				
		**Clean**	**Warning**	**Low**	**Moderately**	**Strongly**	**Clean**	**Warning**	**Low**	**Moderately**	**Strongly**
I	Hg	1.9	1.9	0	1.9	94.3	0	0	5.8	3.9	90.3
	Cd	15.4	7.4	36.6	23.2	17.4					
	As	82.7	11.5	5.8	0	0					
	Pb	71.2	25.0	3.8	0	0					
	Cr(III)	100	0	0	0	0					
II	Hg	1.3	0	5.2	16.5	77.0	0	8.6	5.2	19.2	67.0
	Cd	27.8	16.5	371	12.4	6.2					
	As	56.7	23.7	186	1.0	0					
	Pb	97.9	2.1	0	0	0					
	Cr(III)	100.0	0	0	0	0					
III	Hg	2.0	1.0	3.0	2.0	92.0	2.0	0	6.0	10.0	82.0
	Cd	10.0	4.0	420	11.0	33.0					
	As	73.0	22.0	5.0	0	0					
	Pb	81.0	19.0	0	0	0					
	Cr(III)	99.0	1.0	0	0	0					

Abbreviations: Pi refers to the single-factor pollution index of heavy metals, while PN represents the comprehensive pollution index of heavy metals.

#### 3.1.2. Geo-Accumulation Index

**Figure 2:**
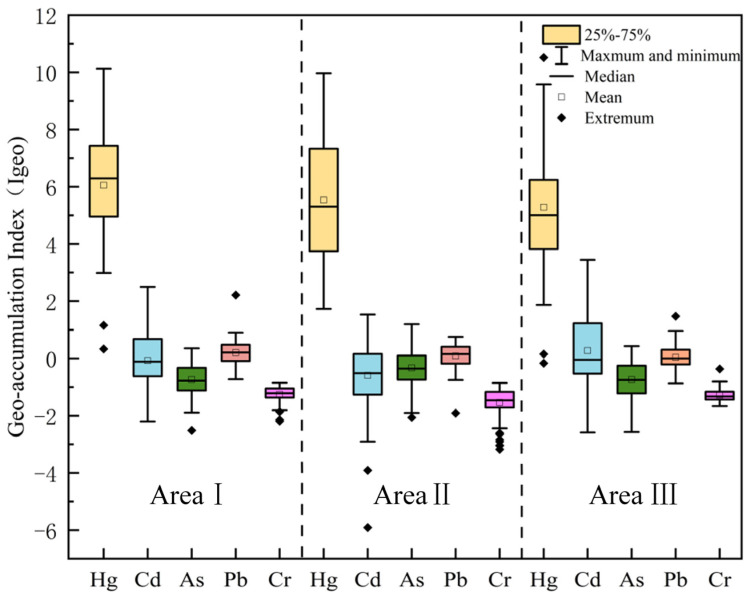
Box plots of land accumulation indices for the study area.

Figure 2 shows the Geo-accumulation Index (Igeo) of the region, illustrating the contamination levels of soil heavy metals across the different areas. In the analysis of the three areas, mercury (Hg) is the most significant pollutant across all areas, with more severe contamination observed in Area I and Area III compared to Area II. The contamination from mercury is most pronounced in Area I, where Igeo values are higher and exhibit considerable variability, suggesting concentrated industrial activities, agricultural pollution, or atmospheric deposition. Extreme pollution values are observed at certain sample points. In comparison, Area III also shows significant mercury contamination, but its distribution is more stable, likely due to more fixed or singular pollution sources in the area. The high levels of mercury contamination are concerning, as they may persist in the soil and potentially enter the ecosystem or food chain, posing long-term risks to both the environment and human health. Cd (cadmium) contamination is also notable in both Area I and Area III. In Area I, the Igeo values are widely distributed, indicating more extensive contamination, while in Area III, the pollution is more concentrated. This pattern may be linked to mining activities, wastewater discharge, or agricultural fertilization practices. Cadmium’s spread is often associated with the long-term accumulation of these human activities, particularly in industrialized areas, where its accumulation in the soil may pose a significant threat to plant growth and the broader ecosystem. In contrast, As (arsenic) and Pb (lead) exhibit relatively low contamination across all areas, particularly in Area II, where Igeo values are almost zero. This suggests that Area II experiences lower pollution pressure, likely due to fewer industrial activities or more effective environmental management and soil types. However, despite their lower contamination levels, the toxicity of arsenic and lead should not be underestimated, as their accumulation in the soil could still have potential environmental impacts, particularly arsenic, which has long-term toxic effects. Cr (chromium) shows no significant pollution across the three areas, with Igeo values close to zero or negative, indicating minimal impact on the soil in these regions. Chromium is often used in alloys or chemicals in industry, and its relatively low presence in the soil suggests that there are fewer pollution sources in these areas, leading to a minimal environmental impact.

Overall, Hg and Cd are the primary soil pollutants in the region, especially in Area I and Area III, where pollution sources are more complex, and contamination levels are higher. In contrast, As, Pb, and Cr show relatively light contamination, but their potential risks should still be monitored. For the areas with high mercury contamination, particularly in Area I, it is crucial to implement effective pollution control measures to prevent long-term environmental and human health consequences.

#### 3.1.3. Spatial Distribution of Heavy Metal Pollution Index in Soil

**Figure 3:**
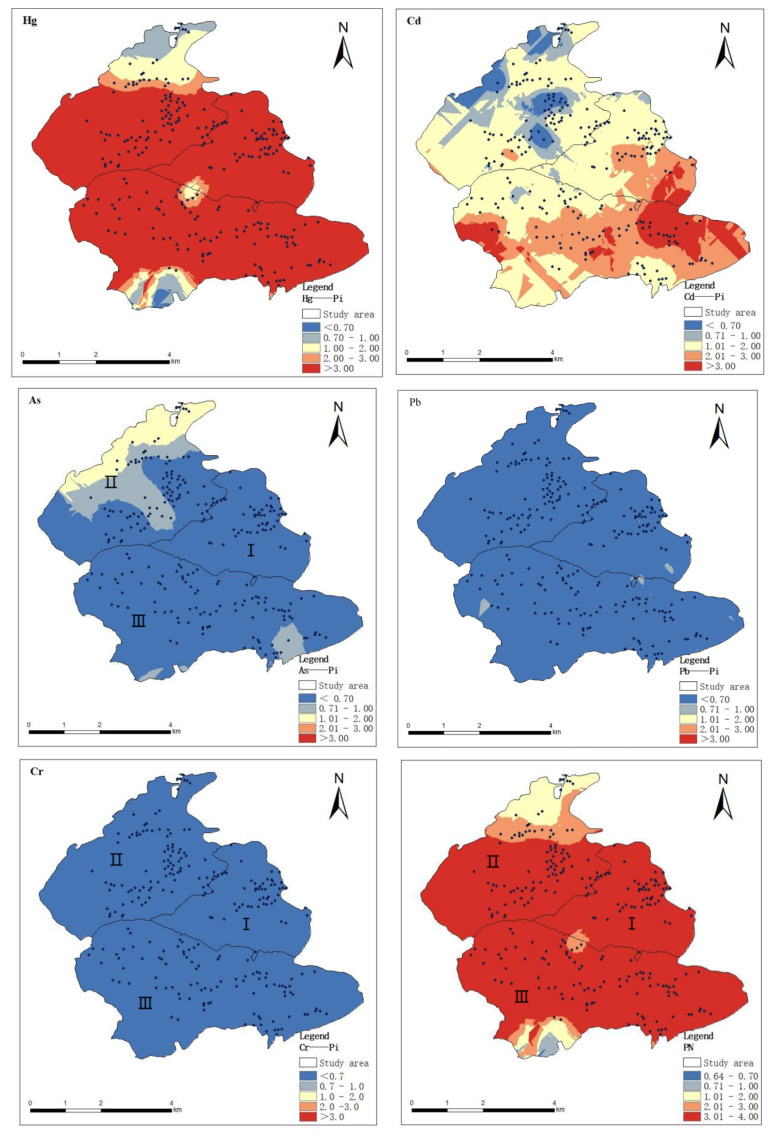
Spatial distribution of soil heavy metal pollution index. I, II, III are three different areas of the study area.

ArcGIS is an important tool for interpreting spatial distribution and environmental monitoring [28], and kriging interpolation, as an effective spatial interpolation method, is capable of continuous unbiased interpolation estimation of discrete variables, which enables the spatial distribution characteristics of heavy metal elements to be visualized. In this study, on the basis of analyzing the spatial structural characteristics of soil heavy metals by combining the parameters of the optimal model of GS+9.0 semi-variable function, the kriging interpolation method of ArcGIS 10.2 was used to draw the spatial distribution maps of soil pollution evaluation of the five heavy metals in the study area, as shown in Figure 3, and the distribution maps consisted of blue, yellow, and red, and the colors gradually transitioned from darker to lighter to indicate that the concentrations were lower than the relevant background values and, respectively, close to and higher than the relevant background values. The darker the red color, the more serious the pollution in the region.

From the visualization results of the soil contamination distribution map (Figure 3), it can be seen that Hg is covered by red color in most of the areas, showing obvious contamination level, which is mainly concentrated in Area I and Area III. This distribution feature may be related to strong industrial activities, agricultural operations, and natural geological factors. The spatial distributions of Cd and As heavy metals in the three areas are more similar, with most of the study area covered by blue color; only a few areas were slightly polluted. Cr in soil, on the other hand, showed no contamination (full blue coverage). The field survey revealed that abandoned mines were present in the areas with high Hg content in the soil. That slag accumulation and direct irrigation with sewage water could be the area’s main sources of Hg contamination. Unlike Hg, there are differences in the spatial distribution patterns of the three heavy metal elements, Cd, As, and Pb. For example, elemental Cd was higher in the south of the study area and relatively lower in the north; elemental As had an obviously high-value area in Area II, while elemental Pb was higher in Area I. The results showed that elemental Cd was found in the south of the study area, while elemental Pb was found in the north. These specific spatial distribution patterns provide an important basis for the subsequent analysis of pollution causes.

### 3.2. CA and PMF

**Figure 4:**
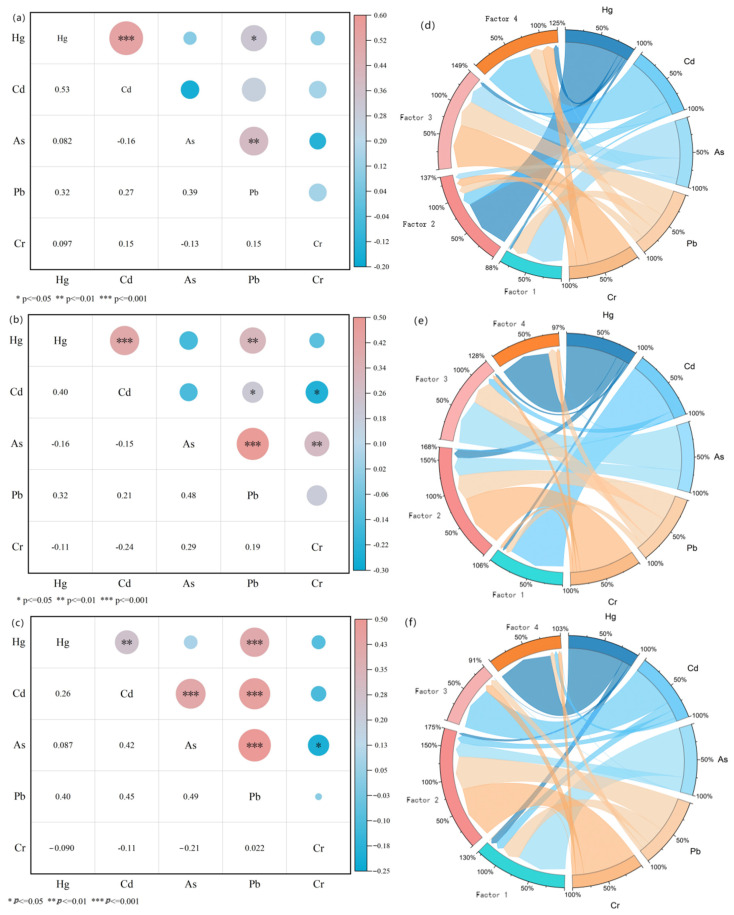
Soil heavy metal source analysis. (**a**–**c**) I, II, and III correlation analysis heatmap; (**d**–**f**) PMFSource resolution identification results; the contribution of the four factors to the heavy metals in I, II, and III, respectively.

The results, including the correlation heat map and PMF outcomes, are displayed in Figure 4 and Table 3. The contribution of different factors to different heavy metals is shown in Table S5.

In Area I, moderate positive correlations (*p* < 0.01) were found between Hg and Cd, and Pb and As, while a weaker positive correlation (*p* < 0.05) was observed between Pb and Hg. The contribution of factor 2 was 27.4%, of which Hg accounted for 83.7%, mainly concentrated in Area I. Analysis of the sources of Hg in the region by previous authors suggests that the main sources of Hg are industrial activities such as non-ferrous metal smelting and mining [29], especially in Guizhou, where there has been a high level of mining activity in the last few decades. Factor 2 was attributed to mining activities [30]. In factor 1, As (58.9%) and Pb (24.1%) were mainly derived from smelting sources, with Pb coming from industrial coal combustion and traffic emissions [31], and As and Pb were enriched in soil through atmospheric precipitation [32]. Chromium contamination in agricultural soils primarily arises from both natural processes, such as rock weathering and human activities [33]; the latter contributes significantly through the discharge of chromium-containing wastewater and solid waste from industries like mining, electroplating, and leather tanning [34]. Given that most agricultural soils are located far from industrial sites and mines, Cr in these soils predominantly originates from soil-forming matrices [35,36]. Furthermore, the chromium concentrations from organic and chemical fertilizers are typically below the natural background levels present in the soil [37]. The agricultural soils examined in this research are located near abandoned mines, and none of the chromium levels in these soils surpassed the local background value, with a coefficient of variation of 29.0% indicating low variability. Still, most of the abandoned mines are located in Area I. Wastewater discharges from mining activities and tailings impoundment affect the soils. Thus, factor 3 can be identified as a combined source stemming from mining activities and parent materials. The main loading factor of factor 4 was element Cd, with 77.9%. Previous studies have found that most of the organic fertilizers and pesticides used in agricultural activities, sewage irrigation, etc., produce Cd deposition in the soil. Cd is also one of the important elements in phosphorus fertilizers required for agricultural production [38]. Therefore, factor 4 can be recognized as an agrarian source in this study.

In Area II, Hg–Cd, Hg–Pb, and Pb–As were positively correlated (*p* < 0.01). PMF analysis showed that factor 3 contributed 25.7% and was mainly composed of Hg (3.2%), Cd (10.7%), As (70.7%), and Pb (39.9%). Atmospheric deposition from highly developed industries in northern China has become a primary contributor to heavy metal concentrations in cultivated soils [39]. Additionally, the buildup of these metals in industrial, mining, and metallurgical Area I is substantial [40]. Factor 2 consists mainly of Cr (84.5%), while Hg (13.4%) and Pb (39.9%) have high loadings on factor 2. The study area is chiefly characterized by red and yellow soils, where the maximum and mean chromium concentrations are below the local baseline values. This implies that chromium primarily stems from the parent material responsible for soil genesis. Therefore, Factor 2 can be ascribed to natural origins. Factor 4 was predominantly influenced by Hg (78.6%). In Area II, untreated tailing ponds allowed tailings to flow into rivers and farmland via rainwater, releasing heavy metals into the soil. Studies have indicated that elevated Hg levels in soils around mining areas are associated with anthropogenic activities related to mining, such as raw ore stockpiling, crushing, and waste disposal [41,42]. Hence, factor 4 is associated with mining origins. Factor 1 was dominated by Cd (87.9%), and the sources included improper use of phosphate fertilizer and pesticides, so factor 1 was of agricultural origin.

Area III demonstrated statistically significant positive correlations (*p* < 0.01) among Hg–Cd, Hg–Pb, and Pb–As. At the same time, Cr was weakly correlated with the other elements, with no significant correlation. This indicates that the sources of chromium contamination and its behavior in the environment differ from those of other heavy metals. Cr will likely originate primarily from specific industrial activities (e.g., electroplating and iron and steel manufacturing) or soil-forming matrices. Meanwhile, Hg, Cd, Pb, and As are more widely available from industry, agriculture, transport, etc. The PMF analysis showed that Factor 1 contributed 0% to Cr and 77.0% and 28.7% to As and Pb, respectively, which were identified as smelting sources; Factor 2 accounted for 82.2% of Cr and was associated with natural sources. Factor 3, dominated by Cd (68.2%), was linked to agricultural activities, while Factor 4, characterized by Hg (85.3%), was associated with mining operations.

Natural sources (soil-forming matrices, tailings impoundments, etc.) and mining sources were the main contributors to pollution in the three regions.

### 3.3. Heavy Metal Health Risk Evaluation

The HQ (Hazard Quotient), THI (Total Hazard Index), CR (Cancer Risk), and TCR (Total Cancer Risk) for Hg, Cr, As, Pb, and Cd across three different regions via oral, respiratory, and dermal pathways in adults and children are illustrated in Table 4.

For the non-carcinogenic health risk to children caused by different exposure pathways of single heavy metals, namely Hg, Cd, As, and Pb elements, followed the path HQ oral > HQ respiratory + dermal, and Cr elements followed a HQ dermal > HQ oral + respiratory path; i.e., the non-carcinogenic risk pathway of Cr elements is mainly dermal contact. The non-carcinogenic risk of the remaining aspects ingested in soil via the mouth is larger. Only Area I’s soil Hg had a mean HQ value greater than 1 for children under the oral-soil pathway, with 17.31% of samples exceeding 1, and the maximum multiplicity of exceedance was 5.07 times. Elemental soil Cd, As, and Cr in all three regions pose varying carcinogenic health risks to children under the oral-soil pathway. The non-carcinogenic risk coefficients (HQ) of different heavy metals for adults and children via the three exposure routes in each region, from high to low, were as follows: oral Hg > Pb > As > Cd > Cr (adults); respiratory: Cd > Hg > As > Pb > Cr; dermal Hg > Cr > As > Pb > Cd; the Hg elemental Hg had the highest risk coefficients for the oral and dermal routes, and the Cd elemental Cd had the highest risk coefficients for the respiratory route of ingestion. The highest risk coefficients were found in the oral and dermal routes for Hg and the respiratory route for Cd. In the three villages of the study area, the magnitude of THI, the total Non-carcinogenic Risk Index caused by different exposure pathways of heavy metals, was shown in the following order for adults: Area I (0.84) > Area II (0.71) > Area III (0.67); for children: Area I (1.17) > Area II (1.21) > Area III (1.14). It can be seen that the non-carcinogenic health risk of children is significantly slightly higher than that of adults. The HQ and THI values of the five heavy metals for adults in all three regions were less than 1, indicating that the health risk due to a single heavy metal does not affect adults in the region. In most cases, children exhibit higher gastrointestinal absorption of certain toxic elements due to their relatively higher respiration rates per unit of body weight [43]. Research has shown that children’s gastrointestinal absorption of toxic metals is significantly higher than that of adults due to their faster metabolic rate and higher body surface area-to-weight ratio. For example, children’s absorption rate for lead can be as high as 50.0%, compared to 10.0% in adults [44]. This heightened absorption rate contributes to the higher non-carcinogenic health risks in children compared to adults. Thus, children are more susceptible than adults to significant health risks arising from environmental heavy metal pollution [45].

Based on the data in Table 4, the carcinogenic health risks (CR) associated with heavy metals (Cd, As, and Cr) in soils across Areas I, II, and III via different exposure pathways (oral ingestion, inhalation, and dermal contact) exhibit notable differences between adults and children. Overall, children exhibit significantly higher total carcinogenic risk (TCR) than adults across all three regions. Particularly, in Area III, the TCR for children reaches 5.8 × 10^−3^, which substantially exceeds the acceptable threshold recommended by the U.S. Environmental Protection Agency (USEPA) (1 × 10^−4^), indicating a severe potential health threat. The TCR for adults in Area III is also elevated at 3.2 × 10^−4^, surpassing the regulatory limit. In contrast, adult TCR values in Areas I and II (3.2 × 10^−5^ and 1.8 × 10^−5^, respectively) remain within acceptable levels, while the corresponding values for children (8.8 × 10^−5^ and 8.9 × 10^−5^) approach the threshold but remain below it. Among the three heavy metals, arsenic (As) is the primary contributors to carcinogenic risk, particularly through oral ingestion. For adults, the oral CR for As in Areas II and III are 1.5 × 10^−4^ and 1.1 × 10^−4^, respectively, both exceeding the acceptable limit. For children in Area III, the oral CR for As is as high as 1.18 × 10^−3^, indicating an alarming level of exposure. In comparison, cadmium (Cd) poses relatively lower carcinogenic risks, with CR values generally in the 10⁻⁶ range or below for both adults and children. However, the oral CR for Cd among children in Area III still reaches 1.04 × 10^−5^ indicating a non-negligible cumulative impact.

Regarding exposure pathways, oral ingestion is the dominant route contributing to carcinogenic risk, followed by dermal contact, while inhalation presents minimal risk across all regions and metals, with CR values consistently in the 10^−8^ to 10^−11^ range. Notably, children exhibit higher CR values across all pathways compared to adults, likely due to their higher metabolic rate, enhanced gastrointestinal absorption, and behavioral factors such as increased hand-to-mouth activity [46]. Children’s exposure to heavy metals like lead and mercury can affect their cognitive development, making them more vulnerable to developmental disorders and learning difficulties than adults.

### 3.4. The Contribution of Heavy Metals

**Figure 5:**
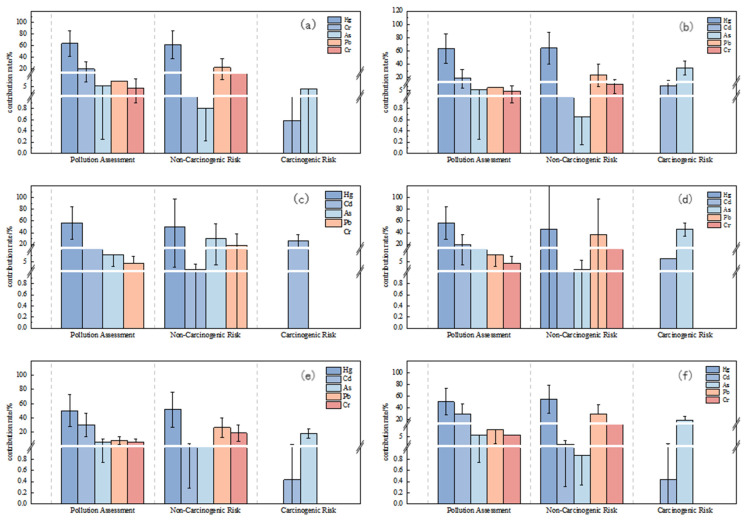
Contribution of soil heavy metals to soil contamination, evaluating non-carcinogenic and carcinogenic risk. (**a**,**b**) Area I—adult and children; (**c**,**d**) Area II—adult and children; (**e**,**f**) Area III—adult and children.

Figure 5 illustrates the varying contributions of five soil heavy metals (Hg, Cd, As, Pb, and Cr) to pollution evaluation, non-carcinogenic health risk, and carcinogenic risk assessments in the study area. Soil Hg had the highest mean contributions to integrated pollution and possible environmental risk across all three regions, with values of approximately 63.7%, 56.7%, and 50.1% for pollution and 96.6%, 95.5%, and 94.9% for ecological risk, respectively.

As shown in Table 5, for mercury (Hg), the non-carcinogenic health risk for adults is mainly contributed by the oral route of intake, with contributions of 85.5%, 84.2%, and 82.8% for Areas I, II, and III, respectively. Inhalation and dermal exposure contributed less. The risk of mercury exposure for children was also dominated by oral intake, with contributions of 84.9%, 83.3%, and 83.1% in the regions, respectively. Thus, the oral route of mercury intake contributed the most to the non-carcinogenic risk for both adults and children. For cadmium (Cd), the exposure risk for adults was mainly manifested through the inhalation route, with contributions of 16.4%, 10.3%, and 33.2% in Areas I, II, and III, respectively. For arsenic (As), the risk for adults was mainly expressed through the inhalation route of exposure, with the highest contribution in Area II (73.2%). Oral intake contributed less, at 13.6%, 14.9%, and 15.7%, respectively. The non-carcinogenic risk for children was mainly contributed by the oral route, especially in Areas II and III, with contributions of 54.2% and 52.3%, respectively.

The contribution of the heavy metals Cd and As to the carcinogenic health risk of adults and children through different exposure pathways varied in different regions. For adults, the carcinogenic risk of Cd was mainly through the dermal exposure route, especially in Areas I, II, and III, where the contribution of the dermal exposure route was 57.9% and 75.5%, respectively. In contrast, children were mainly exposed to cadmium through oral ingestion, which contributed 46.9% and 95.2% in Areas I and II, respectively. In Area III, on the other hand, children’s cancer risk was almost entirely from oral intake. For As, the carcinogenic risk for adults was mainly through the oral intake route, with contributions of 43.1%, 47.2%, and 34.7%, respectively. For children, oral intake almost completely dominated the carcinogenic risk of As, especially in Areas I, II, and III, where the contribution of oral intake was 95.2% and 97.6%, respectively. The pathway of the carcinogenic risk of Cr for adults was more complex, with oral intake pathway, dermal contact, and inhalation pathway.

In summary, there are significant differences in the pathways of health risk between adults and children when exposed to these heavy metals.

## 4. Conclusions

This study reveals the complexity of heavy metal contamination in the soil of mercury mining areas, particularly the significant contamination of mercury (Hg). By applying the Geo-accumulation Index (Igeo), we found that mercury contamination was severe in the study areas, especially in Areas I and III, where the Igeo values were high, indicating that the mercury contamination reached moderate to extreme levels. This result is closely related to the mining and smelting processes in the area, where continuous mining activities as well as wastewater and tailings discharge have exacerbated mercury accumulation in the soil. The results of this study reveal that mercury (Hg) is the predominant pollutant in the Tongren mercury mining region, with high accumulation levels especially in Areas I and III. This aligns with previous research conducted in similar mining regions such as the Xunyang mercury mine in Shaanxi and the Wanshan mine in Guizhou, where Hg accumulation was also shown to dominate the pollution profile due to prolonged mining and smelting activities [47]. The observed high Geo-accumulation Index (Igeo) values and Potential Ecological Risk Index (PERI) for Hg suggest persistent environmental contamination despite the mine having been closed for years. This underscores the long-term legacy impact of mercury mining on soil quality.

This study highlights the complex nature of heavy metal contamination in the Tongren region, with a focus on mining activities and natural sources as the primary contributors to the observed pollution. The results from the PMF (Positive Matrix Factorization) analysis clearly demonstrate that both mining and natural sources jointly contribute to the contamination of heavy metals such as mercury (Hg) and cadmium (Cd). Mining activities, especially those related to historic mercury extraction and other industrial processes, have had a substantial impact on the contamination levels, particularly in Areas I and III. On the other hand, natural sources, including soil-forming parent materials and tailings impoundments, play a significant role in the contamination of chromium (Cr), which is primarily derived from geogenic processes. Our study confirms that mining remains the dominant source of contamination for Hg and Cd, while natural sources are a significant contributor to Cr levels, highlighting the need for differentiated management strategies for pollution control. The combined effect of these two sources underscores the importance of addressing both anthropogenic and geogenic factors in environmental management and health risk assessments [11].

When compared with studies in other regions, such as the copper mine in Jiangxi [48], it becomes evident that the Tongren area presents a more severe ecological risk specifically related to Hg and Cd. Notably, while other sites show dominant contamination by As or Pb, the extreme Hg levels observed here—up to 286 mg/kg—are indicative of unique local geochemical and anthropogenic conditions.

The health risk assessment further reveals critical insights, particularly regarding vulnerable populations. Children exhibited Non-carcinogenic Hazard Indices (THI) exceeding the safety threshold (THI > 1) in all three areas, with oral ingestion of Hg being the main exposure route. These results echo findings from [49], which reported that children’s greater absorption efficiency and behavioral patterns significantly amplify their susceptibility to heavy metal exposure. Additionally, the carcinogenic risk analysis suggests that chromium (Cr) and arsenic (As) pose unacceptable cancer risks to both adults and children, especially in Area III, where the TCR values exceed the upper limit of the acceptable range recommended by USEPA (1 × 10^−4^).

These results highlight the importance of refining pollution prevention strategies in such legacy mining areas. Although the current regulatory framework may help limit new pollution sources, residual contamination in soil continues to pose substantial health threats. Therefore, our findings support the need for comprehensive soil remediation plans, targeted health monitoring for residents—especially children—and long-term environmental surveillance to track changes in heavy metal bioavailability.

## Figures and Tables

**Table 3:** Contribution of different factors to the pollution status of the three regions.

Source	Area I	Area II	Area III
Mining activity	27.4%	15.9%	16.9%
Smelting	3.3%	25.7%	18.1%
Agricultural sources (pesticides; fertilizers)	18.3%	6.1%	9.4%
Natural sources (soil-forming matrices, tailings impoundments, etc.)	51.0%	52.3%	55.6%

**Table 4:** Results of Health Risk Evaluation of Non-Carcinogenic and Carcinogenic Soil Heavy Metals.

Heavy Metals	Ingestion Pathway	Non-Carcinogenic Health Risk					
		Adult			Children		
		I	II	III	I	II	III
Hg	Oral ingestion	0.6	0.5	0.5	1.1	0.9	0.8
	Inhalation	2.6 × 10^−4^	2.2 × 10^−4^	2.01 × 10^−4^	1.2 × 10^−4^	9.7 × 10^−5^	9.3 × 10^−5^
	Dermal contact	3.2 × 10^−2^	0.027	0.024	0.036	0.86	0.027
Cd	Oral ingestion	4.9 × 10^−3^	3.4 × 10^−3^	7.3 × 10^−3^	8.8 × 10^−3^	6.2 × 10^−3^	0.013
	Inhalation	2.1 × 10^−4^	1.4 × 10^−4^	4.9 × 10^−4^	9.5 × 10^−5^	6.7 × 10^−5^	5.5 × 10^−4^
	Dermal contact	6.9 × 10^−4^	4.9 × 10^−4^	1.0 × 10^−3^	9.5 × 10^−5^	5.6 × 10^−4^	1.2 × 10^−3^
As	Oral ingestion	1.8 × 10^−3^	2.3 × 10^−3^	1.7 × 10^−3^	1.2 × 10^−3^	1.6 × 10^−3^	1.2 × 10^−3^
	Inhalation	7.7 × 10^−4^	1.0 × 10^−3^	7.8 × 10^−4^	3.5 × 10^−4^	4.8 × 10^−4^	3.6 × 10^−4^
	Dermal contact	1.1 × 10^−3^	1.4 × 10^−3^	1.1 × 10^−3^	3.2 × 10^−3^	4.3 × 10^−3^	3.2 × 10^−3^
Pb	Oral ingestion	0.10	9.4 × 10^−2^	9.1 × 10^−2^	0.19	0.17	0.16
	Inhalation	8.5 × 10^−6^	7.6 × 10^−6^	7.4 × 10^−6^	3.9 × 10^−6^	3.5 × 10^−6^	3.4 × 10^−6^
	Dermal contact	4.2 × 10^−4^	3.8 × 10^−4^	3.7 × 10^−4^	4.8 × 10^−4^	4.3 × 10^−4^	4.2 × 10^−4^
Cr(III)	Oral ingestion	2.86 × 10^−4^	6.1 × 10^−5^	2.8 × 10^−6^	5.1 × 10^−4^	1.1 × 10^−4^	4.9 × 10^−4^
	Inhalation	8.7 × 10^−6^	6.2 × 10^−7^	8.5 × 10^−6^	2.8 × 10^−4^	1.9 × 10^−5^	2.7 × 10^−4^
	Dermal contact	9.6 × 10^−2^	5.1 × 10^−2^	5.8 × 10^−2^	6.8 × 10^−2^	5.7 × 10^−2^	6.6 × 10^−2^
THI		0.7	0.7	0.6	1.2	1.21	1.14
**Carcinogenic Health Risk**							
Cd	Oral ingestion	1.5 × 10^−6^	1.8 × 10^−5^	3.7 × 10^−5^	7.8 × 10^−6^	4.9 × 10^−6^	1.0 × 10^−5^
	Inhalation	1.7 × 10^−11^	1.2 × 10^−11^	2.5 × 10^−11^	2.7 × 10^−11^	1.7 × 10^−11^	3.6 × 10^−11^
	Dermal contact	5.3 × 10^−9^	6.3 × 10^−8^	1.3 × 10^−7^	2.0 × 10^−6^	3.4 × 10^−8^	7.0 × 10^−8^
As	Oral ingestion	1.4 × 10^−5^	1.5 × 10^−4^	1.1 × 10^−4^	3.1 × 10^−5^	4.2 × 10^−5^	1.18 × 10^−3^
	Inhalation	1.6 × 10^−9^	4.9 × 10^−8^	1.2 × 10^−10^	1.7 × 10^−9^	2.3 × 10^−9^	2.1 × 10^−8^
	Dermal contact	1.5 × 10^−10^	1.6 × 10^−5^	1.1 × 10^−9^	6.1 × 10^−10^	8.2 × 10^−10^	6.2 × 10^−10^
TCR		1.6 × 10^−5^	1.7 × 10^−5^	1.5 × 10^−4^	4.3 × 10^−5^	4.7 × 10^−5^	4.2 × 10^−5^

**Table 5:** Contribution of each heavy metal to non-carcinogenic health risk and carcinogenic risk under different exposure routes.

Heavy Metals	Ingestion Pathway	Non-Carcinogenic Health Risk					
		Adult			Children		
		I	II	III	I	II	III
Hg	Oral ingestion	85.5%	84.2%	82.8%	84.9%	83.3%	83.1%
	Inhalation	21.0%	15.5%	13.5%	14.2%	10.9%	7.2%
	Dermal contact	24.6%	33.7%	28.3%	33.6%	93.2%	27.7%
Cd	Oral ingestion	0.7%	0.6%	1.3%	0.7%	0.6%	1.3%
	Inhalation	16.4%	10.3%	33.2%	11.1%	7.6%	43.4%
	Dermal contact	0.5%	0.6%	1.2%	0.1%	0.1%	1.2%
As	Oral ingestion	0.2%	0.4%	0.3%	0.1%	0.1%	0.1%
	Inhalation	61.2%	73.2%	52.3%	41.3%	54.2%	27.9%
	Dermal contact	0.8%	1.8%	1.3%	2.9%	0.5%	3.3%
Pb	Oral ingestion	13.6%	14.9%	15.7%	14.3%	15.9%	15.5%
	Inhalation	0.7%	0.5%	0.5%	0.5%	0.4%	0.3%
	Dermal contact	0.3%	0.5%	0.4%	0.4%	0.0%	0.4%
Cr	Oral ingestion	0.0%	0.0%	0.0%	0.0%	0.0%	0.0%
	Inhalation	0.7%	0.5%	0.6%	32.9%	27.0%	21.3%
	Dermal contact	73.7%	63.4%	68.7%	62.9%	6.2%	67.4%
**Carcinogenic Health Risk**							
Cd	Oral ingestion	4.6%	5.6%	11.7%	8.8%	5.5%	0.8%
	Inhalation	0.0%	0.0%	0.0%	0.0%	0.0%	0.0%
	Dermal contact	57.9%	0.1%	75.5%	98.9%	62.9%	0.2%
As	Oral ingestion	43.1%	47.2%	34.7%	34.9%	46.9%	95.2%
	Inhalation	2.2%	17.5%	0.2%	1.0%	2.4%	5.4%
	Dermal contact	1.6%	22.0%	0.6%	0.0%	1.5%	0.0%

## Data Availability

The availability of the data used in this study is subject to restrictions. The data were obtained from Hanjiang Eco-Hydraulic (Wuhan) Co., Ltd., and are available to researchers only with proper authorization. Under the terms of the confidentiality agreement, readers may contact the corresponding author to request data access.

## Supplementary Materials

The following supporting information can be downloaded at: https://www.mdpi.com/article/10.3390/toxics13070527/s1, Table S1. Classification of The Geo-accumulation Index (Igeo); Table S2. Classification criteria for soil environmental quality; Table S3. Classification criteria of oil heavy metal Potential Ecological Risk Index [50,51]; Table S4. Toxicology characteristic parameters for the heavy metals; Table S5. Source apportionment for different heavy metals conducted by PMF model.

## Abbreviations

The following abbreviations are used in this manuscript:

Hg	Mercury
Cd	Cadmium
As	Arsenic
Pb	Lead
Cr	Chromium
PMF	Positive definite matrix factor model

## References

[1] Yoon S., Kim D.-M., Yu S., Park J., Yun S.-T. (2023). Metal(Loid)-Specific Sources and Distribution Mechanisms of Riverside Soil Contamination near an Abandoned Gold Mine in Mongolia. J. Hazard. Mater..

[2] Wang Z., Yu Y., Ye T., Fei J., Song X., Peng J., Zhou Y., Wu H. (2022). Distribution Characteristics and Environmental Risk Assessment Following Metal(Loid)s Pollution Incidents at Southwest China Mining Site. Trans. Nonferrous Met. Soc. China.

[3] Zhang Y., Zhou J., Gao F., Zhang B., Ma B., Li L. (2015). Comprehensive Ecological Risk Assessment for Heavy Metal Pollutions in Three Phases in Rivers. Trans. Nonferrous Met. Soc. China.

[4] Johnson D.B., Hallberg K.B. (2005). Acid Mine Drainage Remediation Options: A Review. Sci. Total Environ..

[5] Wang Y., Zhang Z., Li Y., Liang C., Huang H., Wang S. (2024). Available Heavy Metals Concentrations in Agricultural Soils: Relationship with Soil Properties and Total Heavy Metals Concentrations in Different Industries. J. Hazard. Mater..

[6] Huang C., Guo Z., Li T., Xu R., Peng C., Gao Z., Zhong L. (2023). Source Identification and Migration Fate of Metal(Loid)s in Soil and Groundwater from an Abandoned Pb/Zn Mine. Sci. Total Environ..

[7] Su C., Wang J., Chen Z., Meng J., Yin G., Zhou Y., Wang T. (2023). Sources and Health Risks of Heavy Metals in Soils and Vegetables from Intensive Human Intervention Areas in South China. Sci. Total Environ..

[8] Ministry of Ecological Environment of the People’s Republic of China (2014). Nation Soil Contamination Survey Bulletin.

[9] Chen H., Teng Y., Lu S., Wang Y., Wang J. (2015). Contamination Features and Health Risk of Soil Heavy Metals in China. Sci. Total Environ..

[10] Kohzadi S., Shahmoradi B., Ghaderi E., Loqmani H., Maleki A. (2019). Concentration, Source, and Potential Human Health Risk of Heavy Metals in the Commonly Consumed Medicinal Plants. Biol. Trace Element Res..

[11] Doležalová Weissmannová H., Mihočová S., Chovanec P., Pavlovský J. (2019). Potential Ecological Risk and Human Health Risk Assessment of Heavy Metal Pollution in Industrial Affected Soils by Coal Mining and Metallurgy in Ostrava, Czech Republic. Int. J. Environ. Res. Public Health.

[12] Men C., Liu R., Xu L., Wang Q., Guo L., Miao Y., Shen Z. (2020). Source-Specific Ecological Risk Analysis and Critical Source Identification of Heavy Metals in Road Dust in Beijing, China. J. Hazard. Mater..

[13] Huang J., Guo S., Zeng G., Li F., Gu Y., Shi Y., Shi L., Liu W., Peng S. (2018). A New Exploration of Health Risk Assessment Quantification from Sources of Soil Heavy Metals under Different Land Use. Environ. Pollut..

[14] Xue L., Zhao Z., Zhang Y., Liao J., Wu M., Wang M., Sun J., Gong H., Guo M., Li S. (2020). Dietary Exposure to Arsenic and Human Health Risks in Western Tibet. Sci. Total Environ..

[15] Liang J., Liu Z., Tian Y., Shi H., Fei Y., Qi J., Mo L. (2023). Research on Health Risk Assessment of Heavy Metals in Soil Based on Multi-Factor Source Apportionment: A Case Study in Guangdong Province, China. Sci. Total Environ..

[16] Guan Q., Zhao R., Pan N., Wang F., Yang Y., Luo H. (2019). Source Apportionment of Heavy Metals in Farmland Soil of Wuwei, China: Comparison of Three Receptor Models. J. Clean. Prod..

[17] Mehmood A., Aslam Mirza M., Aziz Choudhary M., Kim K.-H., Raza W., Raza N., Soo Lee S., Zhang M., Lee J.-H., Sarfraz M. (2019). Spatial Distribution of Heavy Metals in Crops in a Wastewater Irrigated Zone and Health Risk Assessment. Environ. Res..

[18] Mao C., Song Y., Chen L., Ji J., Li J., Yuan X., Yang Z., Ayoko G.A., Frost R.L., Theiss F. (2019). Human Health Risks of Heavy Metals in Paddy Rice Based on Transfer Characteristics of Heavy Metals from Soil to Rice. CATENA.

[19] De Souza V.B., Hollas C.E., Bortoli M., Manosso F.C., De Souza D.Z. (2023). Heavy Metal Contamination in Soils of a Decommissioned Landfill Southern Brazil: Ecological and Health Risk Assessment. Chemosphere.

[20] Chen L., Ren B., Deng X., Yin W., Xie Q., Cai Z., Zou H. (2024). Potential Toxic Elements (PTEs) in Rhizosphere Soils and Crops under a Black Shale High Geological Background: Pollution Characteristics, Distribution and Risk Assessment. Ecol. Indic..

[21] Wu Z., Zhang L., Xia T., Jia X., Wang S. (2020). Heavy Metal Pollution and Human Health Risk Assessment at Mercury Smelting Sites in Wanshan District of Guizhou Province, China. RSC Adv..

[22] Qin F., Wei C., Zhong S., Huang X., Pang W., Jiang X. (2016). Soil Heavy Metal(Loid)s and Risk Assessment in Vicinity of a Coal Mining Area from Southwest Guizhou, China. J. Cent. South Univ..

[23] Zhao Z., Li S., Li Y. (2024). Determining the Priority Control Factor of Toxic Metals in Cascade Reservoir Sediments via Source-Oriented Ecological Risk Assessment. J. Hydrol..

[24] (2004). Technical Specifications for Environmental Soil Monitoring.

[25] USEPA (2011). Exposure Factors Handbook.

[26] Zheng S., Wang Q., Yuan Y., Sun W. (2020). Human Health Risk Assessment of Heavy Metals in Soil and Food Crops in the Pearl River Delta Urban Agglomeration of China. Food Chem..

[27] Zhou H., Chen Y., Yue X., Ren D., Liu Y., Yang K. (2023). Identification and Hazard Analysis of Heavy Metal Sources in Agricultural Soils in Ancient Mining Areas: A Quantitative Method Based on the Receptor Model and Risk Assessment. J. Hazard. Mater..

[28] Dong B., Zhang R., Gan Y., Cai L., Freidenreich A., Wang K., Guo T., Wang H. (2019). Multiple Methods for the Identification of Heavy Metal Sources in Cropland Soils from a Resource-Based Region. Sci. Total. Environ..

[29] Zhao Z., Hao M., Li Y., Li S. (2022). Contamination, Sources and Health Risks of Toxic Elements in Soils of Karstic Urban Parks Based on Monte Carlo Simulation Combined with a Receptor Model. Sci. Total Environ..

[30] Yan J., Wang C., Wang Z., Yang S., Li P. (2019). Mercury Concentration and Speciation in Mine Wastes in Tongren Mercury Mining Area, Southwest China and Environmental Effects. Appl. Geochem..

[31] Li Y., Zhou S., Jia Z., Liu K., Wang G. (2021). Temporal and Spatial Distributions and Sources of Heavy Metals in Atmospheric Deposition in Western Taihu Lake, China. Environ. Pollut..

[32] Liang J., Feng C., Zeng G., Gao X., Zhong M., Li X., Li X., He X., Fang Y. (2017). Spatial Distribution and Source Identification of Heavy Metals in Surface Soils in a Typical Coal Mine City, Lianyuan, China. Environ. Pollut..

[33] Li X., Zhang J., Ma J., Liu Q., Shi T., Gong Y., Yang S., Wu Y. (2020). Status of Chromium Accumulation in Agricultural Soils Across China (1989–2016). Chemosphere.

[34] Yang Q., Li Z., Lu X., Duan Q., Huang L., Bi J. (2018). A Review of Soil Heavy Metal Pollution from Industrial and Agricultural Regions in China: Pollution and Risk Assessment. Sci. Total Environ..

[35] Wang Y., Zhang L., Wang J., Lv J. (2020). Identifying Quantitative Sources and Spatial Distributions of Potentially Toxic Elements in Soils by Using Three Receptor Models and Sequential Indicator Simulation. Chemosphere.

[36] Liu H., Zhang Y., Yang J., Wang H., Li Y., Shi Y., Li D., Holm P.E., Ou Q., Hu W. (2021). Quantitative Source Apportionment, Risk Assessment and Distribution of Heavy Metals in Agricultural Soils from Southern Shandong Peninsula of China. Sci. Total Environ..

[37] Lv J. (2019). Multivariate Receptor Models and Robust Geostatistics to Estimate Source Apportionment of Heavy Metals in Soils. Environ. Pollut..

[38] Wu Q., Hu W., Wang H., Liu P., Wang X., Huang B. (2021). Spatial Distribution, Ecological Risk and Sources of Heavy Metals in Soils from a Typical Economic Development Area, Southeastern China. Sci. Total Environ..

[39] Peng H., Chen Y., Weng L., Ma J., Ma Y., Li Y., Islam M.S. (2019). Comparisons of Heavy Metal Input Inventory in Agricultural Soils in North and South China: A Review. Sci. Total Environ..

[40] Huang Y., Wang L., Wang W., Li T., He Z., Yang X. (2019). Current Status of Agricultural Soil Pollution by Heavy Metals in China: A Meta-Analysis. Sci. Total Environ..

[41] Zhao B., O’Connor D., Huang Y., Hou R., Cai L., Jin Y., Wang P., Zhang H. (2024). An Integrated Framework for Source Apportionment and Spatial Distribution of Mercury in Agricultural Soil near a Primary Ore Mining Site. Chemosphere.

[42] Zhao B., O’Connor D., Zhang H., Jin Y., Wang Y., Yang X., Hou R., Hou D. (2023). Assessing Mercury Pollution at a Primary Ore Site with Both Ancient and Industrial Mining and Smelting Activities. Environ. Pollut..

[43] Ma W., Tai L., Qiao Z., Zhong L., Wang Z., Fu K., Chen G. (2018). Contamination Source Apportionment and Health Risk Assessment of Heavy Metals in Soil Around Municipal Solid Waste Incinerator: A Case Study in North China. Sci. Total Environ..

[44] Ziegler E.E., Edwards B.B., Jensen R.L., Mahaffey K.R., Fomon S.J. (1978). Absorption and Retention of Lead by Infants. Pediatr. Res..

[45] Tong S., Li H., Wang L., Tudi M., Yang L. (2020). Concentration, Spatial Distribution, Contamination Degree and Human Health Risk Assessment of Heavy Metals in Urban Soils across China between 2003 and 2019—A Systematic Review. Int. J. Environ. Res. Public Health.

[46] Tolins M., Ruchirawat M., Landrigan P. (2014). The Developmental Neurotoxicity of Arsenic: Cognitive and Behavioral Consequences of Early Life Exposure. Ann. Glob. Health.

[47] Zhu D., Wei Y., Zhao Y., Wang Q., Han J. (2018). Heavy Metal Pollution and Ecological Risk Assessment of the Agriculture Soil in Xunyang Mining Area, Shaanxi Province, Northwestern China. Bull. Environ. Contam. Toxicol..

[48] Barkett M.O. (2018). Heavy Metal Contents of Contaminated Soils and Ecological Risk Assessment in Abandoned Copper Mine Harbor in Yedidalga, Northern Cyprus. Environ. Earth Sci..

[49] Lanphear B.P., Hornung R., Khoury J., Yolton K., Baghurst P., Bellinger D.C., Canfield R.L., Dietrich K.N., Bornschein R., Greene T. (2005). Low-Level Environmental Lead Exposure and Children’s Intellectual Function: An International Pooled Analysis. Environ. Health Perspect..

[50] (1989). Risk-Assessment Guidance for Superfund Volume 1 Human Health Evaluation Manual (Part A).

[51] Bussard D. (1989). Exposure Factors Handbook.

