# Supplementary material for: Sources, Distribution, and Health Risks of Heavy Metal Contamination in the Tongren Mercury Mining Area: A Case Study on Mercury and Cadmium
Source: Toxics. 2025 Jun 23;13(7):527. doi: 10.3390/toxics13070527 (PMC12298102; doi:10.3390/toxics13070527)
Supplement: Supplementary file 1 [file toxics-13-00527-s001.zip › toxics-3552847-supplementary.pdf]

Supplement material

Table S1. Classification of The Geoaccumulation Index (Igeo)

Environmental indices	Categoriesndex Method
Not to weakly contaminated	Igeo≤ 0
Weakly to moderately contaminated	0 < Igeo ≤ 1.0
Moderately contaminated	1.0 < Igeo ≤ 2.0
Moderately to strongly contaminated	2.0 < Igeo ≤ 3.0
Strongly contaminated	3.0<Igeo≤4.0
Strongly to extremely contaminated	4.0<Igeo≤5.0
Extremely contaminated	Igeo>5.0

Table S2. Classification criteria for soil environmental quality

Single-factor pollution index	The Nemerow Composite Pollution Index Method	Pollution level	Reference
$P_i \leq 0.7$	PN ≤ 0.7	Clean	
$0.7 < P_i \leq 1.0$	0.7 < PN ≤ 1.0	Early warning	
$1.0 < P_i \leq 2.0$	1.0 < PN ≤ 2.0	Mild pollution	[51]
$2.0 < P_i \leq 3.0$	$2.0 < PN_i \leq 3.0$	Moderate pollution	
$P_i < 3.0$	PN<3.0	Severe pollution	

Table S3. Classification criteria of soil heavy metal potential ecological risk index

Symbol	Meaning	Values		Unit	Reference
		Child	Adult		
IngR	frequency of soil ingestion	200	100	$mg \cdot d^{-1}$	[49]
I_{ac}/I_{aa}	air inhalation rate	10	20	$mg \cdot m^{-3}$	[49]
InhR	breathing frequency	7.5	14.5	$m^3 \cdot d^{-1}$	[49]
BW	average body weight	15.9	56.8	kg	[50]
SA	exposed skin surface area	2800	5700	cm^2	[50]
ED	exposure duration	6	24	a	[50]
PET	particulate matter intake factor	$1.36×10^9$	$1.36×10^9$	$m^3 \cdot kg^{-1}$	[50]
SL	Skin adherence factor	0.2	0.07	$mg \cdot cm^{-2} \cdot d^{-1}$	[50]
ABS	Absorption factor	0.001	0.001	/	[50]
EF	Absorption factor,	350	350	$d \cdot a^{-1}$	[50]
AT	Averaging Time	2190（non-carcinogens）<br>26280（carcinogens）		d	[44]

Table S4. Toxicology characteristic parameters for the heavy metals

Parameters	Metals	Pathway		
		Oral Ingestion[a]	Inhalation[b]	Dermal[c]
R_{fd}	Hg	3.0×10^{-4}	8.57×10^{-5}-	2.10×10^{-5}

 https://doi.org/10.3390/xxxxx

	Cd	1.0×10^{-3}	2.55×10^{-6}-	2.50×10^{-5}-
	As	0.3×10^{-3}	0.123×10^{-3}	0.3×10^{-4}
	Pb	3.5×10^{-3}	3.52×10^{-3}	0.14×10^{-3}
	Cr	0.5	0.75×10^{-4}	0.255×10^{-4}
	Hg	-	-	-
	Cd	6.1	3.8×10^{1}	6.1
SF	As	1.5	1.5×10^{1}	3.6
	Pb	-	-	-
	Cr	5.0×10^{-1}	2.0×10^{1}	3.29×10^{2}

(-)data not available, [a][50], [b][50], [c][50]

Table S5. Source apportionment for different heavy metals conducted by PMF model

Parameters	Metals	Hg	Cd	As	Pb	Cr
Area I	Factor 1	3.4	1.9	58.9	24.2	0.0
	Factor 2	83.7	20.1	8.9	12.6	12.1
	Factor 3	5.1	0.0	32.2	41.8	69.8
	Factor 4	7.8	77.9	0.0	21.5	18.1
Area II	Factor 1	4.8	87.9	0.0	9.3	4.5
	Factor 2	13.4	1.3	29.3	39.9	84.5
	Factor 3	3.2	10.7	70.7	37.7	5.8
	Factor 4	78.6	0.02	0.0	13.1	5.2
Area III	Factor 1	10.9	13.8	77.0	28.7	0.0
	Factor 2	3.8	12.2	22.9	54.8	82.1
	Factor 3	0.0	68.2	0.0	11.2	12.1
	Factor 4	85.2	5.9	0.0	6.4	5.8

Area I: Factor 1(natural sources), Factor 2(mining activities and parent materials), Factor 3(atmospheric deposition), Factor 4(agrarian source)

Area II : Factor 1(natural sources), Factor 2(agriculture), Factor 3(atmospheric deposition), Factor 4(mining activities and parent materials)

Area III : Factor 1(atmospheric deposition), Factor 2(natural origins), Factor 3(agriculture), Factor 4(mining activities and parent materials)
